# Significant Reduction in Bone Density as Measured by Hounsfield Units in Patients with Ankylosing Spondylitis or Diffuse Idiopathic Skeletal Hyperostosis

**DOI:** 10.3390/jcm13051430

**Published:** 2024-03-01

**Authors:** Alexander Swart, Abdelrahman Hamouda, Zach Pennington, Nikita Lakomkin, Anthony L. Mikula, Michael L. Martini, Mahnoor Shafi, Thirusivapragasam Subramaniam, Arjun S. Sebastian, Brett A. Freedman, Ahmad N. Nassr, Jeremy L. Fogelson, Benjamin D. Elder

**Affiliations:** 1 Department of Neurologic Surgery, Mayo Clinic, 200 1st St SW, Rochester, MN 55905, USA; 2 Department of Cardiology, Mayo Clinic, 200 1st St SW, Rochester, MN 55905, USA; 3 Department of Orthopedic Surgery, Mayo Clinic, 200 1st St SW, Rochester, MN 55905, USA

**Keywords:** ankylosing spondylitis, bone mineral density, diffuse idiopathic skeletal hyperostosis, fracture, Hounsfield units, stress shielding

## Abstract

**Background:** Multisegmental pathologic autofusion occurs in patients with ankylosing spondylitis (AS) and diffuse idiopathic skeletal hyperostosis (DISH). It may lead to reduced vertebral bone density due to stress shielding. **Methods:** This study aimed to determine the effects of autofusion on bone density by measuring Hounsfield units (HU) in the mobile and immobile spinal segments of patients with AS and DISH treated at a tertiary care center. The mean HU was calculated for five distinct regions—cranial adjacent mobile segment, cranial fused segment, mid-construct fused segment, caudal fused segment, and caudal adjacent mobile segment. Means for each region were compared using paired-sample *t*-tests. Multivariable regression was used to determine independent predictors of mid-fused segment HUs. **Results:** One hundred patients were included (mean age 76 ± 11 years, 74% male). The mean HU for the mid-construct fused segment (100, 95% CI [86, 113]) was significantly lower than both cranial and caudal fused segments (174 and 108, respectively; both *p* < 0.001), and cranial and caudal adjacent mobile segments (195 and 115, respectively; both *p* < 0.001). Multivariable regression showed the mid-construct HUs were predicted by history of smoking (−30 HU, *p* = 0.009). **Conclusions:** HUs were significantly reduced in the middle of long-segment autofusion, which was consistent with stress shielding. Such shielding may contribute to the diminution of vertebral bone integrity in AS/DISH patients and potentially increased fracture risk.

## 1. Introduction

Ankylosing spondylitis (AS) and diffuse idiopathic skeletal hyperostosis (DISH) are two forms of inflammatory arthritis characterized by the autofusion of multiple vertebral levels. Population-based studies have suggested that axial spondyloarthridities, including AS, affects 2.6–49.0 per 10,000 persons. Patients are generally male (2–4:1 male–female ratio), ≤45 years of age, and present with chronic low back pain. Laboratory data may show signs of ongoing inflammation, and, radiographically, patients have sclerosis of the sacroiliac joints (New York classification) [1]. Risk factors include a HLA-B27 positive status (estimated to be present in up to 80% of advanced cases) and the presence of comorbid autoimmune pathologies, specifically inflammatory bowel disease and psoriasis [1,2]. Of note, there is significant variability in disease burden, ranging from 7.4 per 10,000 in Africa to 31.9 per 10,000 in North America [3]. DISH likewise shows global variability in disease burden (4–30% of the population), being most common in developed countries, though it is argued that this may be due to more frequent radiological examinations occurring in developed countries [4]. Like AS, DISH shows a male predominance (2:1), and onset is in the third to fifth decade of life, though the disease is reported most frequently in patients over the age of 50 [4,5]. Reported comorbidities include type 2 diabetes mellitus, obesity, hypertension, and other features of metabolic syndrome, which may account for its reported higher prevalence in Western populations [4,5]. DISH patients have also been reported to have high levels of growth hormone in the serum and synovial fluid, which may promote osteoblast proliferation and excessive ossification.

The exact pathogenesis promoting these changes is currently unclear, but AS is thought to result from an immunological process, whereas DISH is thought to result from growth factor dysregulation [6,7]. AS is characterized by erosive changes at the corners of the vertebral bodies, followed by the outgrowth of bony spurs known as syndesmophytes in advanced stages [8]. It commonly presents in young patients and is associated with sacroiliac joint ankylosis. Conversely, DISH is characterized by ossification along the anterolateral aspect of contiguous vertebrae and predominately affects an older patient population [9]. Unlike AS, it is also commonly asymptomatic. Regardless, both diseases display ligamentous ossification and bridging syndesmophytes that join multiple segments of the mobile spine, resulting in biomechanics more akin to those of a long bone. However, the erosions and apophyseal joint ankylosis present in AS are not seen in DISH. Additionally, the AS-associated syndesmophytes are slender, vertical, and involve the outer margin of the annulus fibrosis. In comparison, those of DISH have a flowing “candlewax” appearance due to ossification of the anterior longitudinal ligament (ALL). DISH also appears to prefer the thoracic spine, whereas AS appears to involve both the thoracic and lumbar spine [10,11]. In both, marginal bone sclerosis can be accompanied by depletion of the vertebral body cancellous bone [12,13], reflected as reduced bone mineral density (BMD) within the autofused segments. This is potentially reflective of stress shielding within the autofusion, where the reduced loading of the autofused segments limits mechanical stresses on the cancellous bone with resultant remodeling.

BMD is conventionally measured with dual-energy X-ray absorptiometry (DXA). However, DXA overestimates spinal BMD in the setting of degenerative changes occurring in spondylosis and spinal ankylosis, thereby limiting its utility in this patient population [14,15,16,17]. Measuring opportunistic computed tomography (CT)-based Hounsfield units (HU) is an alternative technique for estimating spinal BMD that has gained popularity of late, and it is relatively immune to degenerative changes [17,18,19,20,21,22,23,24,25,26,27]. Evaluation of the relative differences in BMD between autofused and mobile segments of the AS and DISH spine must be thoroughly explored. Documentation of differences in the BMD of mobile and immobile segments helps explain the disproportionate rate of fractures within the fused segments of these two patient populations, as well as the clear biomechanical issues associated with a long-segment spinal fusion. Therefore, the present study aims to compare spinal BMD as estimated by HUs on opportunistic CT to compare vertebral HUs as a surrogate for cancellous bone density between fused and non-fused segments in patients with AS and DISH.

## 2. Materials and Methods

Adults with AS or DISH treated at a single institution comprised of three affiliated tertiary care centers between January 2000 and January 2020 were identified after obtaining institutional review board approval (IRB #18-002622). Preliminary power analysis indicated a 15 HU difference between groups, which could be detected with a sample size of 63 (power 80%, alpha 0.05). Patients were included if they had radiologically confirmed AS or DISH, underwent operative management of a mobile spine fracture, were >18 years of age, had preoperative pan-spine CT imaging, and had ≥1 mobile segments of the spine adjacent to the autofused region. Patients were excluded when the autofusion extended cranially to C2 or caudally to S1, or if they had prior spinal instrumentation. 

For each patient, data were extracted on demographics, medical comorbidities, and baseline BMD estimated by HUs on preoperative CT. The demographics obtained were age, gender, body mass index (BMI), and smoking history. Comorbidities queried included hypertension, type 2 diabetes mellitus (DM), congestive heart failure, end-stage renal disease, prior myocardial infarction, peripheral vascular disease, ascites, disseminated cancer, and chronic obstructive pulmonary disease. The baseline comorbidity burden was quantified using the Charlson Comorbidity Index (CCI) score [28]. 

**Figure 1:**
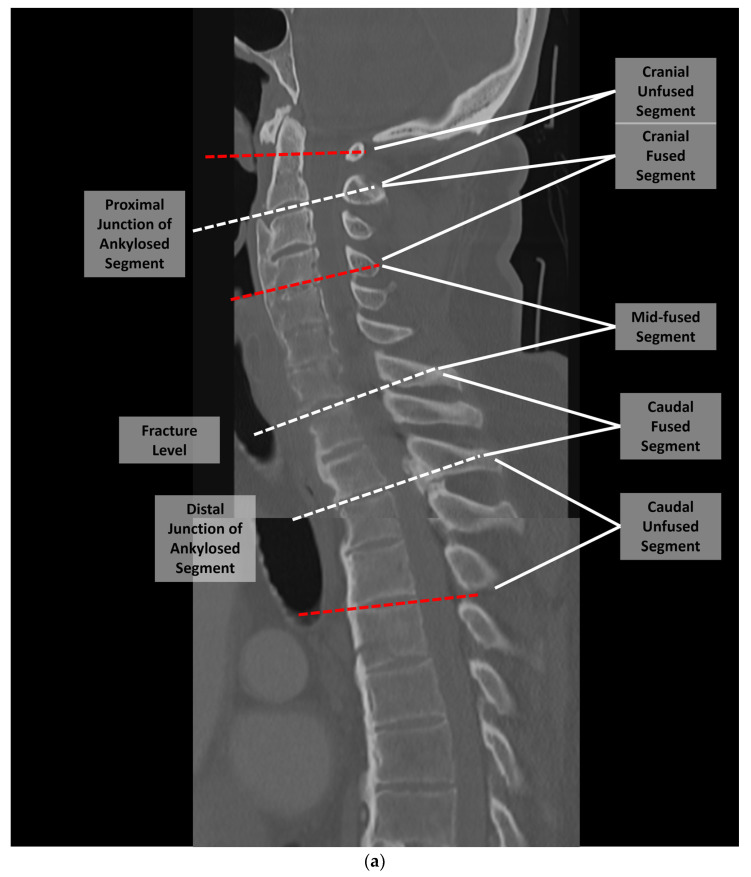

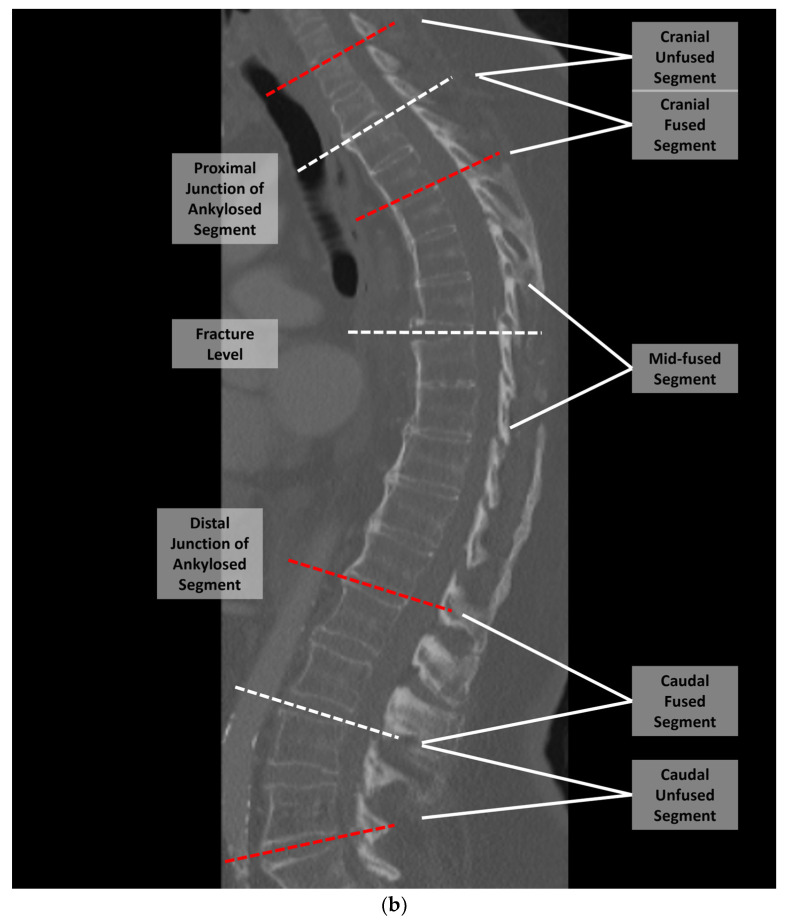

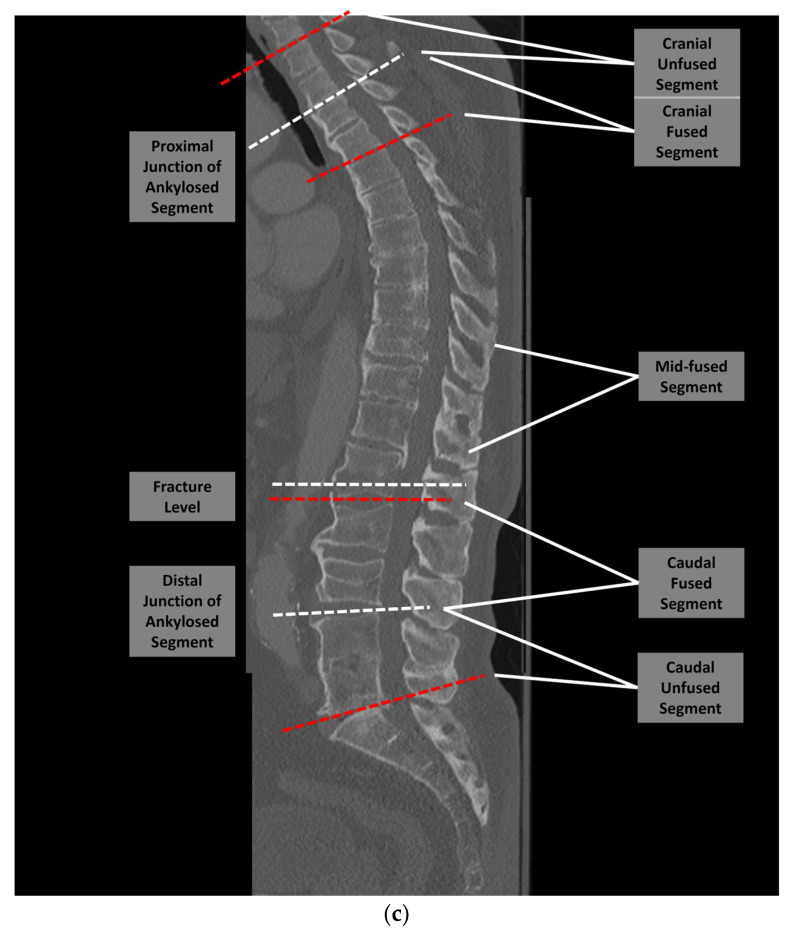
Each patient’s spinal CT was examined, and the regions were divided into the cranial adjacent mobile segment, cranial fused segment, mid-fused segment, caudal fused segment, and caudal adjacent mobile segment. These are illustrated examples of the analysis for patients with (**a**) cervical, (**b**) thoracic, and (**c**) lumbar fractures. For some cervical fractures the ankylosis, extended to the C2/3 junction, in which case the HUs of the cranial unfused segment was the HU average.

**Figure 2:**
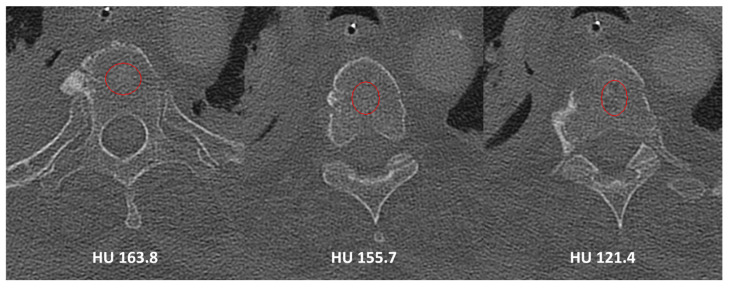
Illustration of measurement of Hounsfield units at cranial, mid-body, and caudal levels of example vertebra (T4 in present example). Red circles indicate the “region of interest” used to measure the HU for the level.

HUs were assessed using preoperative CT scans obtained within 1-year of treatment. Two independent assessors calculated HUs at five spinal segments for each patient: *cranial adjacent mobile segment* (upper instrumented vertebrae [UIV]+1/+2), *cranial fused segment* (UIV/UIV−1), *mid-fused segment*, *caudal fused segment* (lower instrumented vertebrae [LIV]/LIV+1), and *caudal adjacent mobile segment* (LIV−1/−2; Figure 1). For the adjacent mobile segments and cranial/caudal fused segments, HUs were averaged across up to two vertebral bodies (one vertebrae—C2—if autofusion extended to C2/3 junction); in the mid-fused segment, HUs were averaged across up to three vertebral bodies, depending on the length of the autofused segment. As previously described, HU measurements of individual vertebrae were performed on axial images at three locations within each vertebral body (cranial, middle and caudal, Figure 2) [22]. The HU values in the mid-fused segment were further categorized as osteoporotic based on the previously reported HU cutoff of <110 [18,23]. In patients with available DXA results, bone quality was categorized as normal (*t*-score > −1.0), osteopenic (*t*-score −1.0 to −2.5), or osteoporotic (*t*-score < −2.5).

Descriptive statistics were employed to report the distributions of continuous and categorical variables of interest. Continuous variables were presented as mean (±standard deviation [SD]), and categorical variables were described as counts and percentages. The mean HU for each of the five spinal segments were compared using a paired-sample *t*-test to evaluate whether they were statistically different from each other. Hierarchical multiple regression was then performed to examine the relative association of age, gender, BMI, smoking status, and CCI to predict the mid-fused segment’s BMD. Preliminary analyses were performed to ensure no violation of the assumptions of normality, linearity, multicollinearity, and homoscedasticity. An agreement between DXA and HUs in categorizing BMD was assessed using the related-samples McNemar change test. *A priori* statistical significance was established as *p* < 0.05. All statistical tests were performed using SPSS Statistics v28 (IBM Corporation, Armonk, NY, USA).

## 3. Results

Seventy-four patients were identified that met the inclusion and exclusion criteria (mean age 75 ± 11 years, mean BMI of 33 ± 8.2 kg/m^2^, 74% male, Table 1). Thirty-two percent were current or former smokers. Of the 74 patients, 33 had underlying AS (44.5%), and 33 had underlying DISH (44.5%). The remaining eight had elements of both AS and DISH (11%). There were fractures localized to the cervical spine in eight cases, the thoracic spine in 63 cases, and the lumbar spine in three cases. Fracture levels were as follows: C4—1; C5—1; C6—3; C7—3; T2—1; T3—6; T4—2; T5—3; T6—5; T7—3; T8—7; T9—7; T10—10; T11—10; T12—9; L1—2; L2—0; L3—0; L4—1; L5—0. Using the AO thoracolumbar classification system [29] fractures were documented as the following types: A1—8 patients, A2—0 patients, A3—6 patients, A4—0 patients, B1—2 patients, B2—13 patients, B3—40 patients, C—5 patients. 

**Figure 3:**
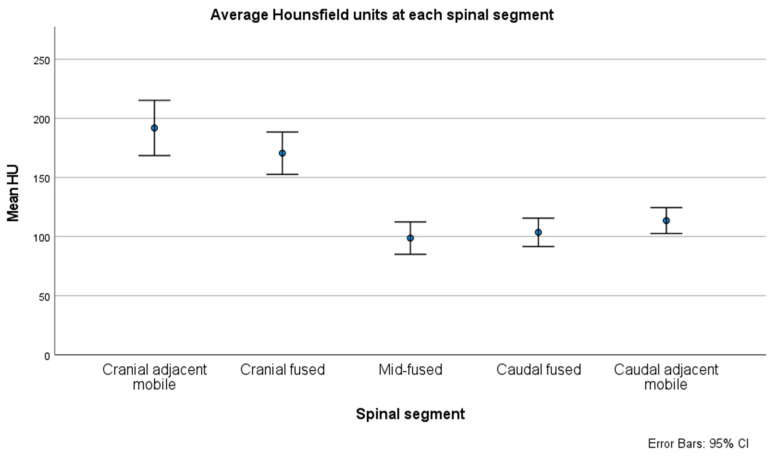
Bone density was lowest in the mid-fused segment and significantly different from the values at all other segments (*p* < 0.001). CI, confidence interval; HU, Hounsfield units.

The mean HU was lowest for the mid-fused segment (100, 95% CI [86, 113]), as demonstrated in Figure 3. Pairwise comparisons between the five regions of interest showed that the HUs of the mid-fused segment were significantly lower than both the cranial (174; 95% CI [157, 192]; *p* < 0.001) and caudal fused segments (108; 95% CI [95, 119]; *p* = 0.05). Similarly, the mid-fused segment HUs were significantly lower than the HUs of both the cranial (195; 95% CI [174, 217]; *p* < 0.001) and caudal adjacent mobile segments (115; 95% CI [104, 126]; *p* < 0.001).

Hierarchical multiple regression showed only age at presentation (β −3.3; 95% CI [−4.3, −2.4]; *p* < 0.001) and history of smoking (β −30.0; 95% CI [−52.3, −7.7]; *p* = 0.009) as independent predictors of mid-fused segment BMD (Table 2). The multivariable model, including age, smoking status, BMI, CCI, and gender, accounted for 42.1% of the variance in the mid-fused segment BMD (R^2^ = 0.421). Once controlled for age, the remaining predictor variables accounted for 10.1% (R^2^ = 0.101).

A subanalysis compared the mid-fused segment HUs between 33 patients with AS alone and 33 with DISH alone (Table 3). Eight patients were excluded from this analysis as they had comorbid AS and DISH. There were no significant differences in gender, age, or BMI between the groups. The presence of DM and current or former smokers was higher in DISH patients (58% *p* < 0.05 and 36% *p* = 0.06, respectively). Mid-fused segment HUs reached 74 in AS patients, significantly lower than that of DISH patients, which was 123 (*p* < 0.001).

## 4. Discussion

AS and DISH are characterized by autofusion of multiple vertebral segments, resulting in a fusion mass with biomechanical properties similar to a long bone. As previously demonstrated in the hip arthroplasty literature [31,32], BMD appears to be positively correlated with the mechanical stresses seen by the bone of interest. Bones subjected to increased biomechanical stresses (i.e., mobile versus fused segments) would be expected to have increased bone density, whereas those seeing lower stresses would be expected to have diminished BMD from stress shielding, as suggested by Wolff’s Law [33]. The present results reflect this hypothesis, with the average HU of vertebrae in the middle of the autofused segment being significantly lower than the HUs of any other segment. Notably, the mean HU of the mid-fusion segment was 100, which is in the osteoporotic range [18], suggesting this bone may be at risk of fragility fractures even in a patient with no other risk factors. Additionally, the average HU of the cranial fused and caudal fused segments were lower than their respective adjacent mobile segment. This concords with prior finite element studies, such as Srinivas and colleagues [34], who studied von Mises stresses on the level adjacent to instrumented fusion. Maximal stresses increased significantly relative to the unfused spine in a manner directly correlated with the number of fused segments; stresses seen in 3-level posterolateral fusion were 20% higher than in the intact spine model.

Load sharing may lead to stress shielding in the long autofused segment, with subsequent loss of bone density and higher fracture risk in the setting of trauma. This study examined the correlation between spinal segment and BMD (as measured by HU) in AS and DISH patients with long-segment autofusion. The results demonstrated a substantial reduction in HUs at the middle segment of the autofusion, with higher HUs at each end of the autofusion and in the adjacent unfused segments. Of note, the difference was greater when comparing cranial autofused and mid-fused segments relative to the mid-fused versus caudal autofused segment comparison. This is likely due to the progressive decrease in average Hounsfield units that is noted with progressively more caudal segments of the human spine. This has been previously described by other groups, most recently Razzouk et al. [35]. Taken together, these findings suggest stress shielding may have significant effects due to vertebral autofusion in AS and DISH patients, helping to explain the increased fracture risk in this patient population.

### 4.1. Basis of Fracture in Ankylosing Spine Disease

Patients with AS and DISH both suffer spinal fractures at a higher rate than the general population [11]. AS patients may experience four-fold the rate of spinal fractures compared to unaffected individuals [36,37,38]. Spinal fractures are relatively common in DISH patients, with as many as 25% of these patients experiencing at least one fracture [39,40]. Importantly, these fractures can occur secondary to trivial trauma, such as a fall from standing height [41], and are considered highly unstable.

The basis for the elevated fracture risk in these patients is thought to be secondary to the altered biomechanics of the ankylosed spine. In a finite element analysis, Nishida et al. considered an adult male T8–sacrum model in the intact state and under conditions where the ALL physical properties were altered to have a Young’s modulus of cortical bone, akin to what is observed in DISH [42]. The T11–12 and T12–L1 segments were also modified with hypertrophic ossifications, and both spines were subjected to ventral and dorsal loading forces. Comparison of maximum von Mises stresses between the normal and DISH models showed significantly higher stresses within the T10–L2 vertebral bodies and interleaving disc spaces of the DISH model in both extension and flexion. With the addition of the hypertrophic ossifications, stresses were selectively elevated at the grooves between ossifications. Stresses were also greatest in the anterior column, with a progressive decline in the middle and posterior columns. This concords with prior examinations of fracture pattern in DISH, which have shown disproportionate involvement of the anterior and middle columns in DISH fractures [41,43,44]. It is noted that most fractures occur between regions of hypertrophic ossification and at junctional regions where autofused segments abut mobile segments, likely due to the generation of stress risers at these points.

In addition to the altered biomechanics, the present results suggest that elevated fracture risk in AS and DISH patients may also result from deterioration of the cancellous density of the autofused bone. With immobilization, the autofused segments likely experience stress shielding and resulting loss of cancellous BMD. Other studies have made similar suggestions, notably recent studies by Lee et al. [45] and Fauny et al. [46]. Both groups analyzed spinal BMD in patients with AS, examining 47 and 73 patients, respectively. In the earlier study, Fauny and colleagues [46] used densitometric scanographic bone evaluation on CT scans, measuring BMD (in HU) on axial slices at the pedicle level. They noted an average HU of 124.3 (L4) to 141.1 (L1) in the lumbar spine with a stepwise decrease in average HU with increasing ankylosis. Those with total ankylosis had an average HU of 29.5% (L1, 111.6 vs. 158.4) to 68.2% lower (L4, 47.7 vs. 150) than patients without syndesmophytes or evidence of ankylosis. Using a threshold for HUs of 145 to identify those at risk of fracture, they noted patients without evidence of autofusion to be roughly half as likely to have BMD, placing them at risk of fracture as compared to those with total ankylosis. Subsequently, in their cohort of 47 males with AS, Lee and colleagues noted a negative correlation between bone bridge formation and BMD (r-value −0.501 to −0.652; *p* ≤ 0.001 for all) [45]. They also noted a correlation between lower BMD and poorer spine mobility, as assessed using the Bath Ankylosing Spondylitis Metrology Index (r −0.387 to −0.570; *p* ≤ 0.01). Though unable to demonstrate a causal relationship, both results suggest that decreased mobility may result in lower BMD and increased fracture risk. To this end, in our cohort, the spinal segment lying in the middle of the fused segment (mid-fused segment) was found to have lower BMD than the adjacent unfused segments, further suggesting stress shielding as the potential etiology for bone integrity loss in ankylosing spine disease.

Apart from advancing age, which is known to correlate with global bone loss, the multiple regression analysis indicated that a history of current or prior tobacco use independently predicted lower spinal BMD [47]. None of the other variables assessed, including BMI, DM, and CCI, were significant predictors of reduced BMD. This result is not surprising, as smoking has been noted as a risk factor for osteoporosis both in men and postmenopausal women [48]. It is thought to act indirectly on bone mass through alteration of body weight, parathyroid hormone-vitamin D axis, adrenal hormones, sex hormones, and increased oxidative stress on bony tissues [47].

Subanalysis demonstrated severely reduced mid-fused segment HUs in AS (74) compared to DISH patients (123), which may help to explain the more severe fractures noted in patients with AS as compared to DISH. A recent study by Chen and colleagues examined 137 patients with AS or DISH presenting with trauma, and they found that AS patients suffered more severe fractures compared to DISH and more frequently underwent surgery for these injuries [44].

### 4.2. Management of Fractures in DISH and Ankylosing Spondylitis

As demonstrated in the present series, despite the predilection for patients with both AS and DISH to experience autofusion of the mobile spine, these patients have notably poor bone quality. Fractures most commonly occur in the middle of the fused segment, with approximately half occurring through an autofused disk space and the other half occurring through a vertebral body [41,43]. While multiple morphologies are possible, the most common in our experience is an extension-distraction type injury, with gapping of the anterior and middle columns [12]. Our approach to preoperative management of this fracture is to place the patient supine in a semi-Fowler’s position, replicating the natural kyphosis seen in the spines of DISH and AS patients. For patients with medical comorbidities or concurrent injuries so severe as to preclude surgery, an external orthosis can be entertained. Given that prior studies have demonstrated a plurality of these lesions to occur in the mid-thoracic or thoracolumbar junction [41,43], this generally involves placement of a thoracolumbosacral (TLSO) brace. For surgical candidates, we place the patient prone on a Wilson frame (Mizuho OSI, Union City, CA, USA) positioned on a flat Jackson table (Mizuho OSI, Union City, CA, USA) to try and restore the native kyphotic alignment. Long-segment instrumented fusion is then performed to stabilize the fracture. Though the present results indicate that the bone quality of the autofused segments is poor, and screws placed at this level are at increased pullout risk, we do not routinely augment these screws. In our experience, such augmentation serves to shift the failure point from the screw–bone interface to the cement–bone interface. Additionally, cement augmentation of the body creates a mismatch in the Young’s modulus of the augmented vertebral body and the adjacent segments. This increases the stress placed on these adjacent segments. Rather, our strategy is to employ multiple points of fixation—three levels above and below the fractured segment—to offload the stress placed on any single fixation point. A prior finite element analysis by Liu et al. found that longer-length constructs have lower maximal stresses on the screws, though the point of maximum stress within the construct remains at the fracture level [49].

### 4.3. Limitations

There are several limitations to the present study. First, patients were selected who presented with significant spinal trauma and may, therefore, represent a high-risk subset of patients with ankylosing spine diseases. The observed differences in HUs between fused and unfused segments may, therefore, be greater than expected in AS and DISH patients. Nevertheless, the observed results agree with prior studies, and it is known that patients with AS and DISH suffer traumatic fractures at rates far higher than age-matched controls. The present results may help to explain this. An additional limitation is that only a subset of patients had a DXA, limiting the comparison between opportunistic CT-based HUs and BMD on DXA. However, an increasing body of literature has begun to suggest that DXA is inadequate for assessing bone quality in patients with ankylosing or degenerative spine diseases. Additionally, there is a growing body of literature suggesting that the conventional DXA cutoffs, i.e., *t*-score > −1.0 for normal bone density, *t*-score −1.0 to −2.5 for osteopenia/low bone density, and *t*-score < −2.5 for osteoporosis, may apply differently to different racial backgrounds. Specifically, some groups have suggested that these conventional DXA cutoffs apply well to Caucasian/white populations, but may apply more poorly to patient of Asian races or other racial backgrounds [50,51,52]. Our study only included patients from a single, multi-site institution, which may limit the generalizability of the findings to other practices. Further studies are warranted with prospective data and a larger sample size. Nevertheless, the present results highlight the need for a better understanding of the biomechanical changes, including diminished bone quality, observed in patients with ankylosing spine diseases. They may highlight the merits of future investigation of prophylactic measures to improve bone health (e.g., anabolic medication administration) in this patient population. Last, the cohort of patients examined in our study comprised patients generally requiring inpatient hospitalization or emergency room consultation for the purposes of recent trauma (low-energy mechanism or otherwise). Consequently, there is no comparison group of patients with ankylosed spines without traumatic injuries. Future studies comparing the present population of patients to those with ankylosed spines without traumatic fracture can help to externally validate the present results. Such a study could confirm whether bone mineral density (as measured by HUs on CT) is lower in the center of autofused segments even among patients with ankylosed spines without concurrent injury. It would additionally allow for the comparison of HUs in the autofused segments between those with and without injury as a means of suggesting a HU value below which patients are at increased risk of traumatic injury.

## 5. Conclusions

In the present multi-site examination of patients with AS or DISH presenting for evaluation of trauma, ankylosed spine segments were demonstrated to have severely reduced spinal BMD relative to mobile segments. Changes were greatest within the middle of the fused segment and consistent with a stress shielding effect, providing an additional potential explanation for the occurrence of fractures within the ankylosed segment of AS and DISH patients following even minor trauma. Though additional validation in larger cohorts is merited, these results may suggest considering prophylactic bone health optimization in these high-risk patients.

## Figures and Tables

**Table 1:** Patient characteristics.

**Gender** (count, %)	
Male	55 (74.3)
Female	19 (25.7)
**Age at presentation** (years, SD)	76 (11.4)
**BMI** (kg/m^2^, SD)	33.4 (8.2)
**Smoking history** (count, %)	
Current/former	23 (31.5)
Never	50 (68.5)
Unavailable	1 (1.4)
**DM** (count, %)	34 (45.9)
**Type of ankylosis** (count, %)	
AS	33 (44.6)
DISH	33 (44.6)
AS/DISH	8 (10.8)
**Fracture Location**	
Cervical	8 (10.8%)
Thoracic	63 (85.1%)
Lumbar	3 (4.1%)
**AO Fracture Morphology** [29,30]	
A0	0 (0%)
A1	8 (10.8%)
A2	0 (0%)
A3	6 (8.1%)
A4	0 (0%)
B1	2 (2.7%)
B2	13 (17.6%)
B3	40 54.1%)
C	5 (6.8%)
**CCI** (score, SD)	5.8 (2.5)

AS, ankylosing spondylitis; BMI, body mass index; CCI, Charlson Comorbidity Index; DISH, diffuse idiopathic skeletal hyperostosis; DM, diabetes mellitus; SD, standard deviation.

**Table 2:** Predictors of mid-fused segment bone mineral density.

Variable	β	*p*-Value	95% CI
Age at presentation (years)	−3.3	<0.001	−4.3 to −2.4
Current or former smoker	−30.0	0.009	−52.3 to −7.7
CCI	−4.8	0.071	−10.1 to 0.4
BMI	1.0	0.142	−0.4 to 2.5
Male gender	9.2	0.460	−15.4 to 33.8

β, beta (effect size); BMI, body mass index; CCI, Charlson Comorbidity Index; CI, confidence interval.

**Table 3:** Subanalysis comparing AS and DISH patients.

Variable	AS	DISH	*p*-Value
Male gender (count, %)	23 (70)	24 (73)	0.79
Age (years, SD)	76.3 (11.8)	74.8 (10.7)	0.6
BMI (SD)	33 (7)	34 (10)	0.53
History of current or former smoking (count, %)	5 (16)	12 (36)	0.06
DM (count, %)	11 (33)	19 (58)	<0.05
CCI (score, SD)	5.9 (2.7)	5.6 (2.5)	0.67
Mid-fused segment HUs (SD)	74 (53)	123 (48)	<0.001

AS, ankylosing spondylitis; BMI, body mass index; CCI, Charlson Comorbidity Index; DISH, diffuse idiopathic skeletal hyperostosis; DM, diabetes mellitus; HU, Hounsfield units; SD, standard deviation.

## Data Availability

The data presented in this study are available on request from the corresponding author. The data are not publicly available due to privacy restrictions.
